# Molecular Characterization of Chicken Anemia Virus Circulating in Chicken Flocks in Egypt

**DOI:** 10.1155/2014/797151

**Published:** 2014-09-15

**Authors:** Mohammed AboElkhair, Alaa G. Abd El-Razak, Abd Elnaby Y. Metwally

**Affiliations:** ^1^Department of Virology, Faculty of Veterinary Medicine, University of Sadat City, Sadat City 32897, Minoufiya, Egypt; ^2^Research Center for Animal Hygiene and Food Safety, Obihiro University of Agriculture and Veterinary Medicine, Inada 2-11, Hokkaido, Obihiro 080-8555, Japan; ^3^Department of Bird and Rabbits Medicine, Faculty of Veterinary Medicine, University of Sadat City, Sadat City 32897, Minoufiya, Egypt; ^4^Animal Health Research Institute, Kafr El Sheikh Provincial Laboratory, Kafr El Sheikh, Egypt

## Abstract

*Introduction*. Although many previous studies reported detection of chicken anemia virus (CAV) in Egypt since 1990, genomic characterization of this circulating CAV has not been published. In the present study, four nucleotide sequences of detected CAV were genetically characterized. *Methods*. These nucleotide sequences were obtained from commercial chicken flocks in two different locations of Egypt during 2010. The target region for sequencing was 675 bp nucleotide of partial coding region of VP1 protein. The nucleotide and deduced amino acid sequences of the detected CAV were aligned and compared to worldwide CAV isolates including commonly used vaccine strains. Phylogenetic analysis of these sequences was also carried out. *Results*. Our results showed that all the Egyptian CAV sequences were grouped in one group with viruses from diverse geographic regions. This group is characterized by amino acids profile ^75^I, ^97^L, ^139^Q, and ^144^Q in VP1. The phylogenetic and amino acid analyses of deduced amino acid indicated that the detected CAV sequences differ from CAV vaccine strains. *Conclusion*. This is the first report that describes molecular characterization of circulating CAV in Egypt. The study showed that the detected CAV, in Egypt are field viruses and unrelated to vaccine strains.

## 1. Introduction

Chicken anemia virus (CAV) is an economically important pathogen with a worldwide distribution. CAV is a small DNA virus with a closed circular, negative, single stranded DNA genome. It belongs to genus* Gyrovirus *of family Circoviridae [1]. The genome consists of three partially overlapping open reading frames encoding three viral proteins: VP1 (51.6 kDa), the major viral capsid protein, VP2 (24 kDa), a novel dual specificity protein phosphatase [2] that also probably acts as scaffolding protein during virion assembly [3], and VP3 (13.6 kDa), also called apoptin, which has been shown to have apoptotic activity in transformed cell lines [4]. VP1 shows the highest nucleotide variability; therefore, it is usually used for genetic characterization and molecular studies of CAV [5, 6].

Infection with CAV constitutes a serious economic threat, especially to the broiler industry and the producers of specific pathogen free (SPF) eggs. The clinical signs are mainly noticed in young chicks of 10–14 days of age, which acquire the infection vertically. Chickens older than 2-3 weeks of age are also susceptible to infection but only develop a subclinical disease evidenced by poor vaccine response [7]. The disease is characterized by aplastic anemia and generalized lymphoid atrophy with concomitant immunosuppression and frequent association with secondary viral, bacterial, parasitic, or fungal infections [7]. Mortalities and morbidities due to CAV infection may reach 55% and 80%, respectively [8].

In Egypt, many previous publications have reported detection of CAV in chicken population (cited in [9–11]); however, genomic characterization from these isolates has not been published. The aim of this study was to identify CAV obtained from farms with problems associated with immunosuppression and to determine their relationship with vaccine and other reference strains.

## 2. Materials and Methods

### 2.1. Samples Collection

Tissue homogenates were collected from Sharkia and Kafr El Sheikh provinces. The homogenates contained thymus loops, bone marrow, bursa of Fabricius, liver, spleen, and intestines samples. Tissue homogenates of Sharkia province were collected from three commercial broiler flocks. The flocks ranged in age from 12 to 35 days representing different breeds and localities in the province. Tissue homogenate of Kafr El Sheikh Province was only collected from one commercial broiler flock aged 25 days. Five liver samples were also collected from a commercial broiler chicken flock aged 30 days from Minoufiya province. All flocks of collected samples were showing clinical signs and lesions indicative of CAV infection [10]. The places, numbers of flocks, and age of flocks of selected samples are listed in Table 1. All samples were stored frozen at −70°C for subsequent DNA extraction.

### 2.2. Sample Preparation

Tissues were homogenized in a mortar with sterile sand and phosphate-buffered saline. Cell debris and sand were eliminated by centrifugation, and the supernatants were collected and stored at −70°C.

### 2.3. DNA Extraction and PCR Amplification

DNA was extracted from the supernatant of liver and tissue homogenates by QIAamp DNA mini kit (Qiagen Inc., Valencia, CA) according to the manufacturer instructions. The oligonucleotide primers 5′-GAC TGT AAG ATG GCA AGA CGA GCT C-3′ and 5′-GGC TGA AGG ATC CCT CAT TC-3′ were used to amplify a 675 bp DNA fragment of Vp1 [12]; from nucleotides 823 to 1498, numbering is corresponding to the Del-Ros strain (GenBank AF313470). The PCR assay was performed in a final volume of 50 *μ*L Reddy-Mix, 18 *μ*L PCR grade water, 1 *μ*L of each primer, and 5 *μ*L template. The amplification was performed under the following conditions: one cycle of initial denaturation step at 95°C for 15 min followed by 30 cycles of 95°C for 1 min, 56°C for 1 min, and 72°C for 1 min representing denaturation, annealing, and extension steps, respectively, and finally one cycle of final extension step at 72°C for 5 min. The amplified products were analyzed using electrophoresis unit. They were loaded to 1% agarose stained by ethidium bromide, visualized under 304 nm ultraviolet (UV Transilluminator, Major Science), and photographed by a gel documentation system using Canon power-shot camera.

### 2.4. Nucleotide Sequence Analysis

Sequencing was performed in both directions with virus specific primers. Sequences were analyzed using BioEdit program [13]. This program was also used to read the sequencing electropherograms to exclude nucleotide ambiguity. The phylogenetic analysis was based on the deduced amino acids of 579 nucleotides encompassing hypervariable region of vp1. The detected sequences were compared with others deposited in GenBank by multiple alignment with the Clustal W included in Bioedit software. Phylogenetic relationships were evaluated by the Neighbor Joining method present in the MEGA version 6 software [14] with 1,000 bootstrap replications. Sequence data were submitted to GenBank with accession numbers KJ955377 to KJ955380 for the Egy-1 CAV to the Egy-4 CAV, respectively.

### 2.5. Attempted Isolation of the Virus

Isolation of CAV from the tissue homogenate of the PCR-positive samples was attempted in embryonated SPF eggs through yolk sac inoculation [7]. Tissue samples were prepared according to [15]. Briefly, the homogenate was mixed with an equal volume of chloroform for 15 min in a shaker. Three times of repeated freezing and thawing were applied and then the homogenate was centrifuged for 20 min at 3000 rpm. The supernatants were used for SPF egg inoculation. Each homogenate was injected in 5 SPF eggs (100 *μ*L/egg). After 14 days, all embryos tissues were homogenized. DNA extraction and PCR amplification were carried out from the supernatant of these tissue homogenates in the same condition as described above. The whole isolation attempt was repeated once.

## 3. Results

### 3.1. Samples and Clinical Signs

All affected birds were at age of 12 to 35 days. All affected flocks showed signs of anemia, generalized weakness, depression, pale comb and wattles, growth retardation, and high mortality rates. The postmortem examination revealed pale and enlarged liver and spleen, mild to severe thymus atrophy, and atrophied bursa of Fabricius.

### 3.2. PCR Amplification

Analysis of PCR amplification of the extracted DNA from tissue samples by agarose gel electrophoresis indicated DNA bands of corrected size as expected with a length of 675 bp. The authenticity of PCR amplification was confirmed by the nucleotide sequencing. All of the liver samples from the flock in Minoufiya province showed negative results. Also, all SPF embryo homogenates were PCR negative.

### 3.3. Nucleotide Sequence Analysis

The four nucleotide sequences of detected CAV displayed a limited diversity. Total nucleotide variation among the sequences ranged from 1 to 29 nucleotides (Table 2(a)). They showed 94.9–99.8% similarity between them. Egy-3 CAV sequence showed the lowest similarity 94.9–96.2% with the other CAV sequences. Egy-1 CAV and Egy-4 CAV showed the highest similarity 99.8%. Compared to isolates from other geographical places around the world, two Egyptian CAV sequences (Egy-1 CAV and Egy-4 CAV) were found to have maximum homology with an Argentinean isolate, Arg0021-3, by 99% and 100%, respectively, whereas the Egy-2 CAV showed 98% homology with a Cameroon strain, CMR09-731, and the Egy-3 CAV was 99% similar to a Cameroon strain, CMR09-485.

### 3.4. Amino Acids Sequence Analysis

The four detected CAV sequences showed 98.9% to 100% identity with each other at the level of amino acid sequence (Table 2 (b)). The maximum difference was between Egy-3 CAV and Egy-1 CAV. These two sequences (Egy-3 CAV and Egy-1 CAV) varied only in two amino acids. The deduced amino acid sequences of the Egyptian CAV sequences and some vaccine strains were aligned. The analysis was carried out to outline the shared amino acid residues between detected viruses and some vaccine strains (Cux-1, Del-Ros, and 26PA) (Table 3). Also, other strains from all over the world were included. The Egyptian CAV sequences showed consensus amino acid sequence at positions 75, 97, 139, and 144 of VP1. At position 22, however, Egy-1 CAV, Egy-2 CAV, and Egy-4 CAV had histidine (H), but Egy-3 CAV had asparagine (N) instead of H. As shown in Table 3, all Egyptian CAV sequences showed sequence difference with regard to vaccine strains. At position 75, the Egyptian CAV had isoleucine (I) instead of valine (V), at position 97, they had leucine (L) instead of methionine (M), at position 139, they had glutamine (Q) instead of lysine (K), and, at position 144, they also had Q instead of glutamic acid (E), N, or aspartic acid (D).

### 3.5. Phylogenetic Analysis

**Figure 1:**
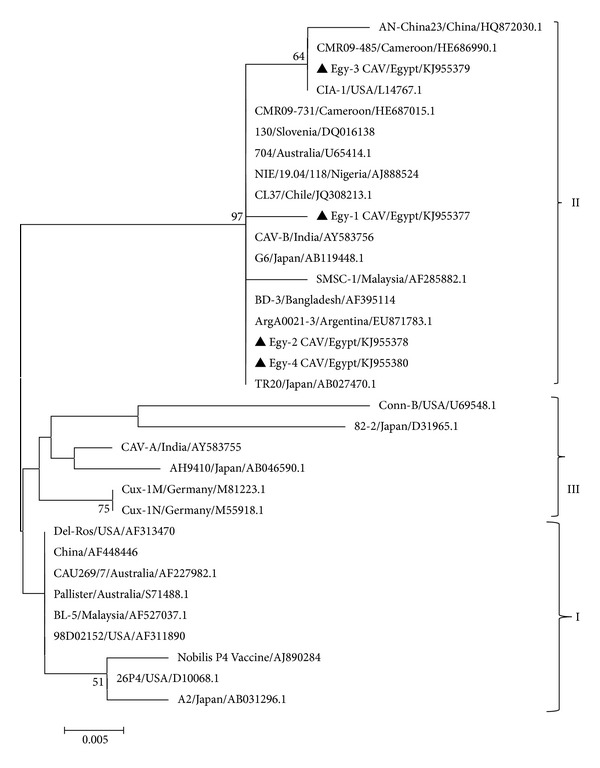
Phylogenetic relationship among 33 different CAV isolates based on partial amino acid sequences of VP1. The tree was constructed by the Neighbor Joining algorithm. Relevant nodes with significant bootstrap values (>50%) over 1000 replicates are indicated. Egyptian detected CAVs are marked in black triangles. The horizontal lines indicate the relative nucleotide distance between samples.

The deduced amino acid sequences of the partial vp1 sequence of Egyptian CAV were compared with those of other sequences deposited in the GenBank. All detected CAVs in Egypt were grouped together in one group (Figure 1). The topology of the phylogenetic tree revealed the presence of three groups that were defined in this study as I, II, and III. The tree topology also showed that the Egyptian sequences were related to different sequences such as NIE/19.04/118/Nigeria, CMR09-731 and CMR09-485/Cameroon, CL37/Chile, CAV-B/India, AN-China 23/China, C1A-1/USA, Arg0021-3/Argentina, and BD-3/Bangladesh which were classified as group II according to [16]. However, different commonly used vaccine strains, for example, Nobilis P4, Del-Ros, 26PA, and Cux-1, grouped with other sequences in groups I and III (Figure 1).

## 4. Discussion

It was reported that CAV spread among chicken in Egypt since the early 1980s when several outbreaks occurred in many breeds [9]. The presence of CAV was confirmed by detection of both CAV antibodies and genome in both meat and egg type chicken flocks [9–11]. However, the molecular characterization from these viruses has not been published. Currently, it is so important to characterize circulating CAVs circulating in Egypt to improve methods of virus control and to determine the relationship of circulating CAV with vaccine strains and other CAV strains. Particularly, a large number of isolates have been fully or partially sequenced. These isolates were obtained in many countries, for example, Bangladesh, Brazil, China, Malaysia, Slovenia, the United States [1],and Argentina [6]. Also, a number of CAV sequences have been reported in African continent, for example, Nigeria [5], Central African Republic, and Cameroon [17], and more recently from South Africa [18]. Comparison of all sequence data indicates that these isolates can be divided into 3 or 4 distinct groups [1]. Vp1 gene sequence is commonly used to determine the relationship of different CAV isolates due to the fact that most of the amino acid substitutions between isolates lie in vp1 gene and more specifically in the N-terminal half of vp1 gene [6, 19]. Moreover, a hypervariable region spanning from amino acid positions 139 to 151 in Vp1 was identified [20]. Islam et al. [16] found that five of 16 commonly variable amino acid positions of the whole vp1 fall within this small region. Therefore, in the present study, we used partial sequencing of vp1 including hypervariable region as a tool to study the molecular characterization of Egyptian CAV sequences. Although the Egyptian detected CAVs showed up to 5.1% difference at the nucleotide sequence level (Table 2(b)), they are almost identical at the amino acid level. This indicated that they differed only by silent mutations.

According to a previous study [16], CAV isolates can be grouped into three different groups based on the amino acid residues at positions of 75, 97, 139, and 144 of amino acid sequence of VP1 protein. In the current study, all detected CAV sequences showed the same amino acid profiles ^75^I, ^97^L, ^139^Q, and ^144^Q. Furthermore, the phylogenetic analysis indicated that all detected Egyptian CAV sequences belong to group II based on Islam et al. classification [16]. The members of this group are from very diverse geographic origins. Also, most of strains isolated from African continent are included in this group.

Interestingly, Egy-3 CAV has a unique amino acid substitution at position 22 which is similar to CIA-1 and AN-China 23 isolates. It had N instead of H at this position. van Santen et al. [21] considered this residue to be important for distinguishing of CAV strains.

It was suggested that the genetic grouping of CAV strains might be related to different biological properties of these strains. Renshaw et al. [20] suggested that ^139^Q and ^144^Q could affect the rate of replication or spread of infection in cultured cells. They have proved that presence of these two amino acids is associated with decreased rate of spread in cell culture. Based on this finding, all detected Egyptian CAVs could have a reduced ability to spread in cell culture.

Also, all detected Egyptian CAVs had threonine in position 89 instead of alanine. It was suggested that this substitution is associated with attenuation [5]. Others [22], however, suggested that the previous mutation should be combined with ^75^I, ^125^L, ^141^L, and ^144^E to produce attenuation. No one of Egyptian detected CAV sequences displayed this combination.

Although the evidence of use of live attenuated CAV vaccines occurring in the Egyptian poultry industry is not readily available, the topology of the phylogenetic tree showed that the CAV Egyptian sequences are in a distant relationship with different vaccine strains commonly used, for example, Nobilis P4, Del-Ros, and Cux-1. All Egyptian CAV sequences are included in group II, whereas vaccine strains are located in groups I and III.

Failure of isolation of CAV in SPF eggs may be due to the low virus concentration in the inoculum or due to the presence of CAV antibodies in the inoculated SPF eggs. It was reported that SPF eggs might contain CAV antibodies [1]. Also, the failure of detection of CAV DNA in livers of Minoufiya chicken flock could be due to inability of the used primers to amplify the proposed CAV because of mutations within the primer location, as we used in this study only one pair of primer set. It was proposed that mutation in location of the primer could lead to loss of detection of some CAV strains [5].

There are some CAV sequences from Egypt (unpublished report) available in the GenBank with accession numbers HM460879.1 to HM460883.1. However, these sequences shared only around 124 nucleotides of VP1 with our sequence. So, the alignment with our sequences was difficult.

## 5. Conclusion

This is the first report that describes the molecular characterization of circulating CAV in Egyptian poultry farms. Although the number of samples was limited, our phylogenetic analysis revealed that CAV strains detected in Egypt were in one group that showed similarity to several strains all over the world, particularly to those circulating in African and South American continents. The results presented in this study clearly showed that the detected CAV sequences belong to field viruses and are not vaccine strains.

## Figures and Tables

**Table 1:** Samples collected for the current study.

Province	Numbers of the flocks	Type of samples	Age of the flock
Sharkia	Three	Tissue homogenates	12–35 days
Kafr El Sheikh	One	Tissue homogenate	25 days
Minoufiya	One	Liver	30 days

**Table:** (a) Nucleotide sequence difference count matrix

	Egy-1 CAV	Egy-2 CAV	Egy-3 CAV	Egy-4 CAV
Egy-1 CAV	ID			
Egy-2 CAV	19	ID		
Egy-3 CAV	29	22	ID	
Egy-4 CAV	1	18	28	ID

**Table:** (b) Amino acid sequence identity matrix

	Egy-1 CAV	Egy-2 CAV	Egy-3 CAV	Egy-4 CAV
Egy-1 CAV	ID			
Egy-2 CAV	99.4%	ID		
Egy-3 CAV	98.9%	99.4%	ID	
Egy-4 CAV	99.4%	100%	99.4%	ID

**Table 3:** Amino substitution in VP1 sequence of CAV isolates.

Isolate	Amino acid positions									
	**22**	48	**75**	83	**97**	125	**139**	141	**144**	157
Consensus	H	A	V	I	M	I	K	Q	Q	V
**Egy-1 CAV/Egypt**	*·*	*·*	**I**	*·*	**L**	*·*	**Q**	*·*	*·*	*·*
**Egy-2 CAV/Egypt**	*·*	*·*	**I**	*·*	**L**	*·*	**Q**	*·*	*·*	*·*
**Egy-3 CAV/Egypt**	**N**	*·*	**I**	*·*	**L**	*·*	**Q**	*·*	*·*	*·*
**Egy-4 CAV/Egypt**	*·*	*·*	**I**	*·*	**L**	*·*	**Q**	*·*	*·*	*·*
Cux-1 M Germany	*·*	*·*	*·*	*·*	*·*	*·*	*·*	*·*	D	*·*
Cux-1 N Germany	*·*	*·*	*·*	*·*	*·*	*·*	*·*	*·*	D	*·*
Del-Ros/USA	*·*	*·*	*·*	*·*	*·*	*·*	*·*	*·*	N	*·*
ConnB/USA	*·*	*·*	*·*	*·*	*·*	*·*	*·*	*·*	E	*·*
26PA/USA	*·*	*·*	*·*	*·*	*·*	*·*	*·*	*·*	E	M
SMSC-1/Malaysia	*·*	*·*	I	*·*	L	*·*	*·*	*·*	*·*	*·*
CIA-1/USA	N	*·*	I	*·*	L	*·*	Q	*·*	*·*	*·*
A2/Japan	*·*	*·*	*·*	*·*	*·*	*·*	*·*	E	E	M
AF448446/China	*·*	*·*		*·*	*·*	*·*	*·*	*·*	E	*·*
BD-3/Bangladesh	*·*	*·*	I	*·*	L	*·*	Q	*·*	*·*	*·*
NIE/19.04/118/Nigeria	*·*	*·*	I	*·*	L	*·*	Q	*·*	*·*	*·*
Isolate704/Australia	*·*	*·*	I	*·*	L	*·*	Q	*·*	*·*	*·*
ArgA0021-3/Argentina	*·*	*·*	I	*·*	L	*·*	Q	*·*	*·*	*·*
AN-China 23/China	N	*·*	I	*·*	L	*·*	Q	*·*	*·*	*·*
CMR09-731/Cameroon	*·*	*·*	I	*·*	L	*·*	Q	*·*	*·*	*·*
CL37/Chile	*·*	*·*	I	*·*	L	*·*	Q	*·*	*·*	*·*
